# Utility of red cell distribution width as a diagnostic and prognostic marker in non-small cell lung cancer

**DOI:** 10.1038/s41598-020-72585-4

**Published:** 2020-09-24

**Authors:** Bin Song, Pengchong Shi, Jianhong Xiao, Yanfang Song, Menglu Zeng, Yingping Cao, Xianjin Zhu

**Affiliations:** 1 Department of Respiratory Medicine, Affiliated Mindong Hospital of Fujian Medical University, 89 Heshan Road, Fuan, 355000 Fujian China; 2 Department of Clinical Laboratory, Fujian Medical University Union Hospital, 29 Xinquan Road, Fuzhou, 350001 Fujian China; 3 Department of Clinical Laboratory, Affiliated People Hospital of Fujian University of Traditional Chinese Medicine, 602 Bayiqi Road, Fuzhou, 350001 Fujian China

**Keywords:** Cancer, Biomarkers

## Abstract

An increasing number of studies have indicated that red blood cell distribution width (RDW) may be a novel biomarker for the diagnosis and prognosis of various malignancies. However, to date, data on the association of RDW with non-small cell lung cancer (NSCLC) are unclear. Our present study aimed to explore the value of RDW in NSCLC patients. A total of 338 NSCLC patients, 109 small cell lung cancer (SCLC) patients, and 302 healthy participants were retrospectively analyzed between January 2016 and December 2018. In the present study, we found that RDW was significantly increased in NSCLC patients. Receiver-operating characteristic (ROC) analysis showed that the area under the ROC curve (AUC) of RDW was 0.753 in discriminating NSCLC patients from healthy participants, the optimal cut-off value of RDW was 12.95, and the specificity and sensitivity were 76.33% and 76.16%, respectively. Further analysis found that RDW can enhance the diagnostic performance of Cyfra21-1 and NSE in discriminating NSCLC patients from healthy participants or SCLC patients. Among NSCLC patients, RDW was significantly correlated with TNM stage, T stage, N stage, M stage, and Cyfra21-1, indicating that RDW may be helpful for predicting the prognosis of NSCLC patients. Our findings suggest that RDW can be used as an auxiliary marker for the diagnosis and prognosis of NSCLC.

## Introduction

Lung cancer, regarded as the 1st fatal cancer in the whole world, is also one of the most common cancers in China^1^. Non-small cell lung cancer (NSCLC) is the most common type of lung cancer and accounts for approximately 80% of all diagnosed lung cancer patients. Unfortunately, most of the NSCLC patients have metastasis at the time of diagnosis^1,2^. Although there are many treatments for NSCLC, including surgery, radiotherapy, and chemotherapy, these treatments bring limited improvements in the long-term survival of NSCLC patients due to the high incidence of recurrence and distant metastasis^2^. Mortality from NSCLC could be reduced by timely screening. Until recently, X-rays, computed tomography (CT) scans, and lung biopsies have been used to diagnose lung cancer, but various limitations restrict their clinical application, such as low sensitivity, high expense or invasive^3,4^. Therefore, to improve treatment strategies, the investigation of simple and readily available biomarkers is urgently needed for the clinic.

Recently, many studies have reported the relationships between various hematological parameters and the clinical diagnosis and treatment process of cancer patients, such as the neutrophil-to-lymphocyte ratio (NLR)^5^, platelet distribution width (PDW)^6,7^, and platelet-to-lymphocyte ratio (PLR)^8^. Red blood cell distribution width (RDW), one of the routine blood indexes, indicates the variability of red blood cell size within a blood sample. In the past, RDW was mainly used for the differential diagnosis of anemia. As an inexpensive and easy-to-measure marker, RDW is gradually attracting attention and being used more and more widely in the clinic^9–12^ . Emerging evidence suggests that RDW plays an important role in tumor diagnosis and prognosis^13^, such as renal cell cancer^14^, breast cancer^15^, ovarian cancer^16^, colorectal cancer^17^, esophageal cancer^18^, endometrial cancer^19^, and gastric cancer^20^.

However, to date, data about the association of RDW with NSCLC are still scarce. In 2013, Yasuko et al. first found that RDW is significantly associated with clinical cancer stage and higher RDW values suggested that lung cancer patients will have poor clinical outcomes^21^. In 2014, Richard et al. found that RDW plays a significant role in determining potentially curative resection in NSCLC patients^22^. In 2016, Mehmet et al. found that RDW levels are significantly and negatively correlated with survival rate^23^. Recently, two reports suggested that RDW is an independent predictor of worse prognosis in lung cancer^24,25^ . These limited reports suggest that RDW may be helpful in predicting prognosis in lung cancer, but there are few studies about the role of RDW in the diagnosis of NSCLC. Additionally, the relationships between histological types of lung cancer and RDW have not been clarified in some studies^21^. Thus, further investigations are needed to analyze the significance of RDW in NSCLC.

In this study, we assessed RDW values to analyze the significance of RDW in diagnosing NSCLC. Moreover, the ability of RDW to indicate prognosis in NSCLC patients was evaluated by analyzing the correlation between clinicopathological features and RDW levels. Our findings suggest that RDW can be used as an auxiliary marker for the diagnosis and prognosis of NSCLC.

## Results

### RDW levels are increased in NSCLC patients

**Figure 1:**
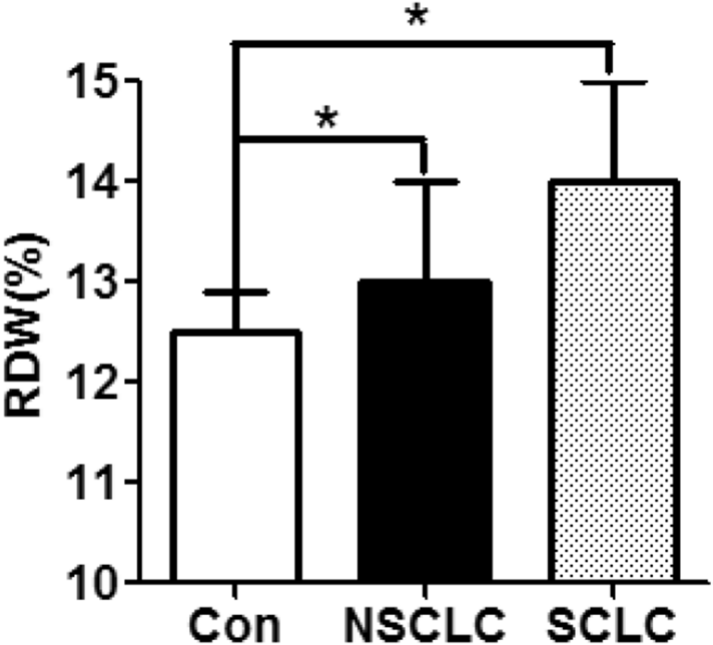
RDW levels in the NSCLC, SCLC, and control groups. RDW levels in NSCLC patients (n = 338), SCLC patients (n = 109), and healthy controls (n = 302) were tested by hematology analyzer. Data are expressed as median and interquartile range. *p < 0.05.

The RDW levels of 338 NSCLC patients, 109 small cell lung cancer (SCLC) patients, and 302 healthy participants were analyzed in this study. The results showed that the RDW levels were significantly higher in the NSCLC group [13.0 (IQR 13.0–14.0)] and the SCLC group [14.0 (IQR 12.85–15.0)] than in the control group [12.5 (IQR 12.18–12.90)] (*P* < 0.05). However, no difference was found in terms of RDW between NSCLC and SCLC patients (Fig. 1). Our data demonstrated that the levels of RDW are increased in NSCLC patients.

### RDW is a useful blood marker to help diagnose NSCLC

**Figure 2:**
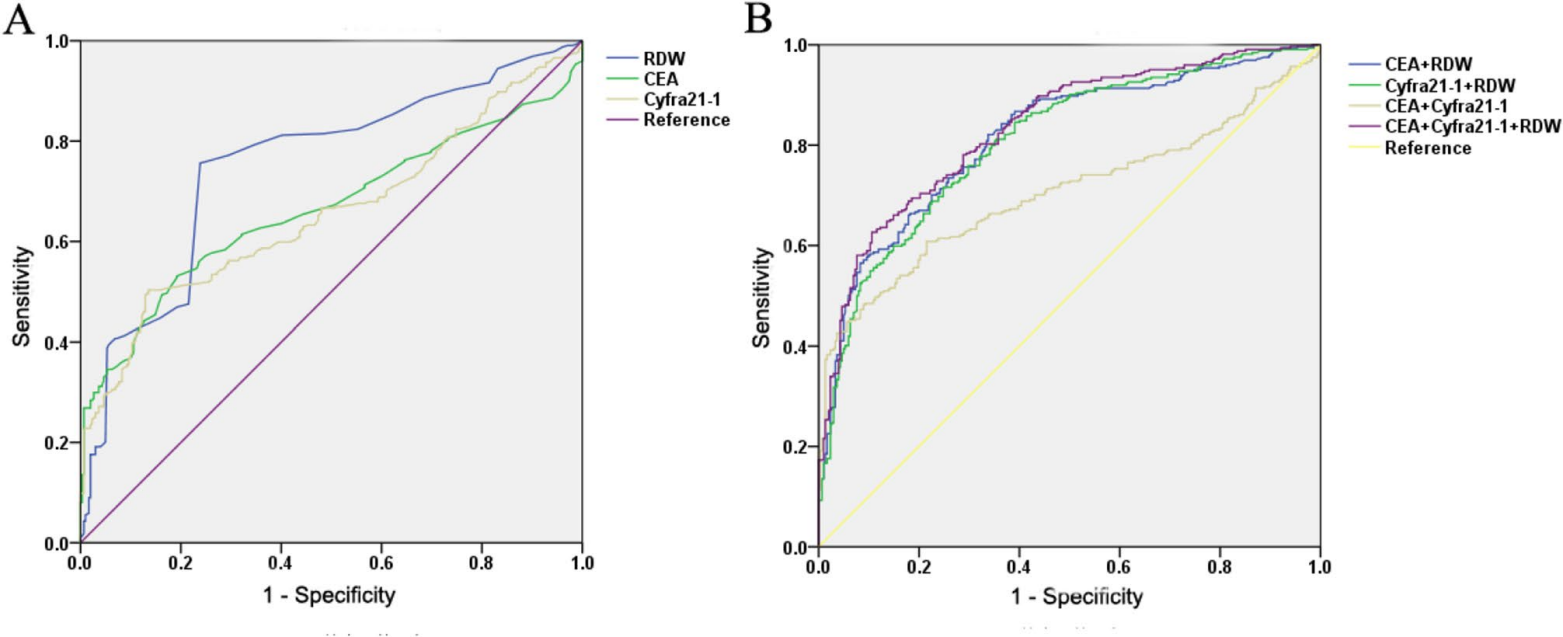
ROC analysis based on RDW for NSCLC diagnosis. (**A**) ROC analysis of value of RDW alone, CEA alone, and Cyfra21-1 alone for NSCLC diagnosis. (**B**) ROC analysis of value of combined detecting RDW, CEA and Cyfra21-1 for NSCLC diagnosis.

ROC curve analysis was used to analyze the power of RDW in the differential diagnosis of NSCLC patients and healthy participants. The results showed that the AUC of RDW was 0.753 (Fig. 2 and Table 1). When the cut-off value of RDW was 12.95, the sensitivity and specificity were 76.33% and 76.16%, respectively (Table 1).

**Table 1:** Diagnostic value of RDW alone, CEA alone and Cyfra21-1 alone and combined detecting for NSCLC diagnosis.

Variables	AUC	Cut off	Sensitivity (%)	Specificity (%)	95% confidence interval	
					Upper limit	Lower limit
RDW	0.753	12.95	76.33	76.16	0.721	0.797
CEA	0.665	5.00	34.32	94.04	0.620	0.705
Cyfra21-1	0.657	3.30	50.31	86.42	0.614	0.700
RDW + CEA	0.816		82.10	66.20	0.783	0.850
RDW + Cyfra21-1	0.808		71.60	75.20	0.775	0.842
CEA + Cyfra21-1	0.705		60.80	78.50	0.663	0.746
RDW + CEA + Cyfra21-1	0.834		62.70	89.40	0.803	0.865

Serum CEA and Cyfra21-1 are common tumor markers in the clinic, but they cannot be widely used in the diagnosis of NSCLC because they lack specificity^26^. In this study, we found that RDW can enhance the diagnostic sensitivity of CEA and Cyfra21-1(Table 1). Importantly, in the differential diagnosis of patients with NSCLC and healthy participants, the AUC values of CEA alone and Cyfra21-1 alone were 0.665 and 0.657, respectively. When CEA, Cyfra21-1, and RDW were combined for detection, the AUC values for CEA + RDW and Cyfra21-1 + RDW were 0.816 and 0.808, respectively, which were significantly higher than those for CEA + Cyfra21-1, CEA alone and Cyfra21-1 alone. When combined detection of lung cancer serum markers (CEA and Cyfra21-1) and blood markers (RDW) was applied, the AUC value was further increased, which was 0.834 (Fig. 2 and Table 1). In brief, these results indicated that RDW can enhance the diagnostic performance of CEA and Cyfra21-1 in the diagnosis of NSCLC.

**Figure 3:**
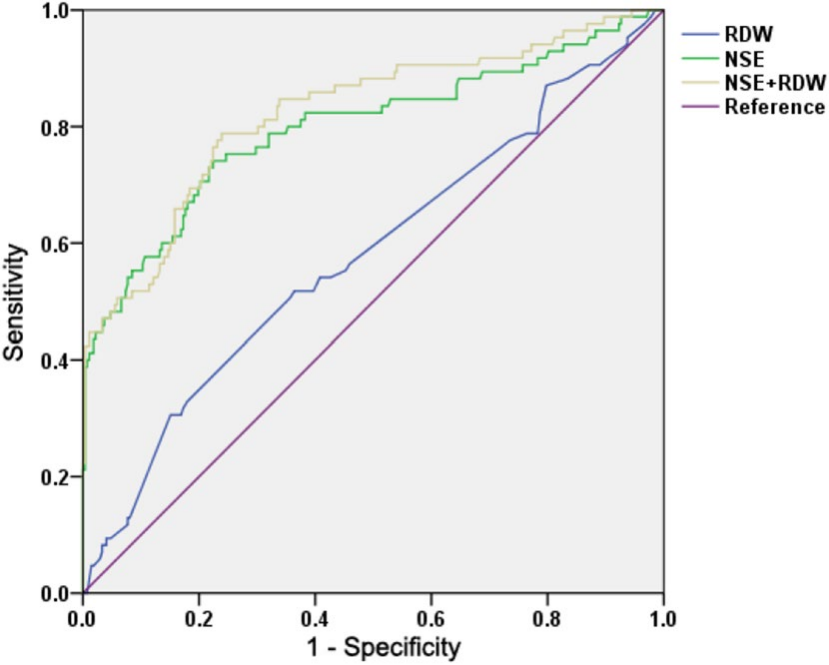
ROC analysis of value of RDW alone and NSE alone, and the combination in differential diagnosis between NSCLC and SCLC. RDW levels in 272 NSCLC patients and 85 SCLC patients were analyzed by ROC curve analysis (NSE levels of some patients is missing).

NSCLC and SCLC are different in terms of their clinical course and response to treatment; thus, it is very important to differentially diagnose NSCLC and SCLC. NSE, a serum tumor marker, is widely used in SCLC patient diagnosis^27^. Next, the results of ROC curve analysis showed that the AUCs of RDW and NSE were 0.581 and 0.798, respectively. When the combined detection of RDW and NSE was applied, the AUC was further increased to 0.824 (Fig. 3 and Table 2), suggesting that RDW alone did not perform well, but can slightly enhance the diagnostic performance of NSE in the differential diagnosis of NSCLC and SCLC.

**Table 2:** Diagnostic values of RDW alone and NSE alone, and combined detecting in differential diagnosis between NSCLC and SCLC.

Variables	AUC	Cut off	Sensitivity (%)	Specificity (%)	95% confidence interval	
					Upper limit	Lower limit
RDW	0.581	14.95	30.60	84.90	0.508	0.653
NSE	0.798	16.30	75.30	72.10	0.736	0.861
RDW + NSE	0.824		78.80	72.10	0.768	0.880

### Relationships between RDW levels and clinicopathological characteristics in NSCLC patients

The patients were categorized according to the RDW cut-off value, which was 12.95%. The correlations between RDW levels and clinicopatholotical characteristics are shown in Table 3. RDW levels were associated with TNM stage, T stage, M stage, N stage, and Cyfra21-1. However, no significant difference in age, sex, histological type, neutrophils, C-reactive protein (CRP), procalcitonin (PCT), or albumin (ALB) was shown in NSCLC patients (Table 3).

**Table 3:** Relationship between RDW and clinical characteristics of NSCLC patients.

Variables	Total	RDW ≤ 12.95	RDW > 12.95	*p*
		n (%)	n (%)	
Sample size	338	80 (23.6)	258 (76.4)	
**Gender**				0.146
Male	196	52 (26.5)	144 (73.5)	
Female	142	28 (19.7)	114 (80.3)	
**Age**				0.151
< 60	158	43 (27.2)	115 (72.8)	
≥ 60	180	37 (20.5)	143 (79.5)	
**Histological type**				0.138
Adenocarcinoma	263	60 (22.8)	203 (77.2)	
Squamous cell carcinoma	65	15 (23.1)	50 (76.9)	
Others	10	5 (50.0)	5 (50.0)	
**TNM stage**				**0.000**
Early (I + II + IIIa)	220	37 (16.8)	183 (83.2)	
Advance (IIIb + IV)	118	43 (36.4)	75 (63.6)	
**pT stage**				**0.000**
T1	145	20 (13.8)	125 (86.2)	
T2	75	17 (22.7)	58 (77.3)	
T3	49	17 (34.7)	32 (65.3)	
T4	69	26 (37.7)	43 (62.3)	
**pN stage**				**0.004**
N0	181	29 (16.0)	152 (84.0)	
N1	22	7 (31.8)	15 (68.2)	
N2	70	25 (35.7)	45 (64.3)	
N3	65	19 (29.2)	46 (70.8)	
**pM stage**				**0.041**
M0	257	54 (21.0)	203 (79.0)	
M1	81	26 (32.1)	55 (67.9)	
**CEA**				0.135
<5	222	47 (21.2)	175 (78.8)	
≥5	116	33 (28.4)	83 (71.6)	
Cyfra21-1*				**0.008**
< 3.3	161	29 (18.0)	132 (82.0)	
≥ 3.3	163	50 (30.7)	113 (69.3)	
**Neutrophile**				0.094
≤ 7.00	279	71 (25.4)	208 (74.6)	
> 7.00	59	9 (15.3)	50 (84.7)	
CRP*				0.067
≤ 8	48	6 (12.5)	42 (87.5)	
> 8.0	35	10 (28.6)	25 (71.4)	
PCT*				0.847
≤ 0.05	25	9 (36.0)	16 (64.0)	
> 0.05	18	7 (38.9)	11 (61.1)	
**ALB**				0.961
≤ 35	64	15 (23.4)	49 (76.6)	
> 35	274	65 (23.7)	209 (76.3)	

*Data of some patients is missing. Bold indicates a statistically significant.

## Discussion

Our findings showed that RDW levels were increased in NSCLC patients and that increased RDW levels can enhance the diagnostic performance of Cyfra21-1 in distinguishing NSCLC patients from healthy participants. Moreover, RDW is closely related to prognostic factors in NSCLC patients, such as tumor stage and tumor markers. Our results showed that RDW may be a useful biomarker in assisting diagnosis and predicting the outcome of NSCLC.

For decades, RDW has been routinely used as a useful index for the differential diagnosis of anemia. In recent years, RDW has attracted notable attention^7^. Various studies have found that RDW is a simple, robust, and convenient biomarker in various malignancies^13^. However, reports on the relationship between RDW and NSCLC are still scarce^21–23^, and further studies are still needed to investigate the role of RDW in NSCLC.

In the current study, the results showed that the levels of RDW were increased in NSCLC patients, which is consistent with the finding of previous report^21–23^. ROC curve analysis showed that RDW was an convenient marker for differentiating NSCLC patients and healthy participants with a cut-off value of 12.95%, sensitivity of 76.33%, and specificity of 76.16%. Cyfra21-1 and NSE, two important serum tumor markers, are often used for the differential diagnosis of lung cancer^26^. However, the low specificity of Cyfra21-1 and NSE limit their clinical application in lung cancer screening. Our results showed that RDW can improve the diagnostic ability of Cyfra21-1 and NSE, suggesting that combining the detection of RDW and Cyfra21-1 or NSE may be a better marker for discriminating NSCLC patients from healthy participants or SCLC patients. To our knowledge, our study is the first to report the clinical utility of the combination of RDW and Cyfra21-1 or NSE for the differential diagnosis of patients with NSCLC.

Next, we investigated whether increased RDW levels might be a potential biomarker for predicting the prognosis of NSCLC patients, and the results showed that reduced RDW was associated with TNM stage, T stage, M stage, N stage, and Cyfra21-1 in NSCLC patients. In contrast, Yasuko et al. reported that RDW is positively correlated with the clinical cancer stage of lung cancer^21^. NSCLC and SCLC have different clinical courses and responses to treatment. However, Yasuko et al. did not analyze the relationships between RDW and NSCLC or SCLC^21^. In addition, the inconsistent findings may be due to the selected populations, small sample sizes, and different RDW cut-off values^21^. Therefore, we need to provide more data to make clarify the associations between RDW and NSCLC. Considering that TNM stage, T stage, M stage, N stage, and Cyfra21-1 are strong prognostic factors for NSCLC, we hypothesize that RDW can be used as a potential prognostic factor in NSCLC patients.

The mechanisms explaining our findings are unclear, but there are some possible explanations. First, some reports have suggested that chronic inflammation plays a significant role in the development and progression of NSCLC, and inflammatory cells release various signaling molecules, which may affect the synthesis or activity of erythropoietin, thus impairing red blood cell maturation and causing immature red blood cells to enter the blood flow^28^. At the same time, inflammation can also lower red blood cell survival, thus resulting in the mixing of red blood cell volumes in peripheral circulation^29^. Previous studies have shown that there is a positive relationship between RDW and inflammatory markers^30^. Second, cancer growth leads to nutritional deficiency, which weakens red blood cell maturation and causes a increased RDW. In this study, we analyzed the relationship between RDW and inflammatory markers (neutrophils, CRP, and PCT) and malnutrition markers (ALB) and found that RDW levels were increased in NSCLC patients with high levels of inflammation markers (neutrophils, CRP, and PCT), but these result were not statistically significant. In addition, RDW levels were not associated with serum ALB levels in NSCLC patients. Therefore, we speculate that RDW is increased and that the relationship between the pathological features and RDW of NSCLC patients may be only partly related to the inflammatory response in NSCLC patients, and the main reasons that may affect RDW levels in NSCLC patients need further study.

Several limitations of the present study remain to be resolved. First, our study was a single-institution, retrospective, relatively small sample size study, which may lead to bias in sample selection and analysis. Second, our study did not assess the association between RDW values and overall survival in NSCLC patients, which limits the generalization of our findings. Therefore, the clinical role of RDW in NSCLC needs to be further verified by other research centers.

Overall, our study indicated that RDW might be used as an auxiliary marker for the diagnosis and prognosis of NSCLC.

## Subjects and methods

### Study subjects

We retrospectively reviewed patients newly diagnosed with lung cancer from January 2016 to December 2018. The exclusion criteria of patients were as follows: anemia, hematologic diseases, and active inflammation. Finally, 338 NSCLC patients [196 male and 142 female, median age (interquartile range age): 61 (53–67) years], 109 SCLC patients [95 male and 14 female, median age (interquartile range age): 64 (57–68) years], and 302 healthy participants [171 male and 131 female, median age (interquartile range age): 60 (55–65) years] were included. The clinicopathological characteristics and laboratory data of the patients were obtained by screening the hospital medical records system. This research was approved by the ethics committee of the Fujian Medical University Union Hospital. Informed consent was obtained from all participants included in the study. All methods were carried out in accordance with relevant guidelines and regulations.

### Laboratory analysis

Peripheral blood samples were collected prior to the start of treatment. Routine blood tests were performed with a Beckman Coulter LH 780 automated hematology analyzer (Beckman Coulter, Brea, CA, USA), the normal range of neutrophils was less than or equal to 7.0× 10^9^/L. The serum carcinoembryonic antigen (CEA), cytokeratin 19 fragments (Cyfra21-1), neuron-specific enolase (NSE), C-reactive protein (CRP), and procalcitonin (PCT) levels were determined by a Cobas 6000 Analyzer (Roche Diagnostics, Basel, Switzerland). According to the manufacturer’s instructions, the normal range of CEA is less than 5 ng/mL, the normal range of CYFRA21-1 is less than 3.3 ng/mL, the normal range of NSE is less than 16.3 ng/mL, the normal range of CRP is less than or equal to 8 mg/L, and that for the normal range of PCT is less than or equal to 0.05 ng/mL. Serum albumin (ALB) was detected with a Beckman DXC800 Biochemical analyzer (Beckman Coulter, Brea, CA, USA), and the normal range of the ALB is more than or equal to 35 g/L.

### Statistical analysis

Statistical analyses were performed using SPSS software version 21.0 (SPSS Inc., Chicago, IL, USA). RDW values are summarized as the median (interquartile range, IQR), and the Mann–Whitney U-test was used to determine the differences between the two groups. ROC curve analysis was performed to assess the diagnostic value of RDW in NSCLC patients. The optimal cut-off value for RDW was determined by the Youden index (Youden index = specificity + sensitivity − 1). The categorical variables are presented as the numbers of patients and percentages, and the Chi-square test was used to compare the differences in incidence of abnormal RDW data between the different groups. All statistical tests were considered statistically significant at a p-value < 0.05.

## References

[1] Chen W (2016). Cancer statistics in China, 2015. CA Cancer J. Clin..

[2] Reck M (2014). Metastatic non-small-cell lung cancer (NSCLC): ESMO Clinical Practice Guidelines for diagnosis, treatment and follow-up. Ann. Oncol..

[3] Tanoue LT, Tanner NT, Gould MK, Silvestri GA (2015). Lung cancer screening. Am J Respir Crit Care Med.

[4] Usman Ali M (2016). Screening for lung cancer: a systematic review and meta-analysis. Prev. Med..

[5] Mei Z (2017). Prognostic role of pretreatment blood neutrophil-to-lymphocyte ratio in advanced cancer survivors: a systematic review and meta-analysis of 66 cohort studies. Cancer Treat. Rev..

[6] Guo F, Zhu X, Qin X (2018). Platelet distribution width in hepatocellular carcinoma. Med. Sci. Monit..

[7] Salvagno GL, Sanchis-Gomar F, Picanza A, Lippi G (2015). Red blood cell distribution width: a simple parameter with multiple clinical applications. Crit. Rev. Clin. Lab. Sci..

[8] Li B (2018). Platelet-to-lymphocyte ratio in advanced cancer: review and meta-analysis. Clin. Chim. Acta.

[9] Goyal H (2017). Prognostic significance of red blood cell distribution width in gastrointestinal disorders. World J. Gastroenterol..

[10] Zhang L (2017). The important role of circulating CYFRA21-1 in metastasis diagnosis and prognostic value compared with carcinoembryonic antigen and neuron-specific enolase in lung cancer patients. BMC Cancer.

[11] Yazici P, Demir U, Bozkurt E, Isil GR, Mihmanli M (2017). The role of red cell distribution width in the prognosis of patients with gastric cancer. Cancer Biomark..

[12] Parizadeh SM (2019). The diagnostic and prognostic value of red cell distribution width in cardiovascular disease; current status and prospective. BioFactors.

[13] Montagnana M, Danese E (2016). Red cell distribution width and cancer. Ann. Transl. Med..

[14] Zyczkowski M (2017). The Relationship between red cell distribution width and cancer-specific survival in patients with renal cell carcinoma treated with partial and radical nephrectomy. Clin. Genitourin. Cancer.

[15] Huang DP, Ma RM, Xiang YQ (2016). Utility of red cell distribution width as a prognostic factor in young breast cancer patients. Medicine.

[16] Qin Y (2017). The value of red cell distribution width in patients with ovarian cancer. Medicine.

[17] Song Y (2018). Clinical usefulness and prognostic value of red cell distribution width in colorectal cancer. Biomed. Res. Int..

[18] Chen GP, Huang Y, Yang X, Feng JF (2015). A nomogram to predict prognostic value of red cell distribution width in patients with esophageal cancer. Mediators Inflamm..

[19] Kemal Y (2015). The value of red blood cell distribution width in endometrial cancer. Clin. Chem. Lab. Med..

[20] Wei TT (2017). Relationship between red blood cell distribution width, bilirubin, and clinical characteristics of patients with gastric cancer. Int. J. Lab. Hematol..

[21] Koma Y (2013). Increased red blood cell distribution width associates with cancer stage and prognosis in patients with lung cancer. PLoS ONE.

[22] Warwick R (2014). Preoperative red cell distribution width in patients undergoing pulmonary resections for non-small-cell lung cancer. Eur. J. Cardiothorac. Surg..

[23] Kos M (2016). Evaluation of the effects of red blood cell distribution width on survival in lung cancer patients. Contemp. Oncol. (Pozn).

[24] Ma C, Wang X, Zhao R (2019). Associations of lymphocyte percentage and red blood cell distribution width with risk of lung cancer. J. Int. Med. Res..

[25] Toyokawa G, Shoji F, Yamazaki K, Shimokawa M, Takeo S (2019). significance of the red blood cell distribution width in resected pathologic stage I nonsmall cell lung cancer. Semin. Thorac. Cardiovasc. Surg..

[26] Sone K (2017). Predictive role of CYFRA21-1 and CEA for subsequent docetaxel in non-small cell lung cancer patients. Anticancer Res..

[27] Harmsma M, Schutte B, Ramaekers FC (2013). Serum markers in small cell lung cancer: opportunities for improvement. Biochim. Biophys. Acta..

[28] Jelkmann W (1998). Proinflammatory cytokines lowering erythropoietin production. J. Interferon Cytokine Res..

[29] Kiefer CR, Snyder LM (2000). Oxidation and erythrocyte senescence. Curr. Opin. Hematol..

[30] Lippi G (2009). Relation between red blood cell distribution width and inflammatory biomarkers in a large cohort of unselected outpatients. Arch. Pathol. Lab. Med..

